# Risk of active tuberculosis development in contacts exposed to infectious tuberculosis in congregate settings in Korea

**DOI:** 10.1038/s41598-020-57697-1

**Published:** 2020-01-28

**Authors:** Shin Young Park, Sunmi Han, Young-Man Kim, Jieun Kim, Sodam Lee, Jiyeon Yang, Un-Na Kim, Mi-sun Park

**Affiliations:** Division of TB Investigation, Korea Centers for Disease Control and Prevention, Osong, Republic of Korea

**Keywords:** Tuberculosis, Tuberculosis, Risk factors, Risk factors

## Abstract

Contact investigation is an important and effective active case-finding strategy, but there is a lack of research on congregate settings in countries with an intermediate incidence. This study determined the incidence of and risk factors for tuberculosis (TB) development after exposure in congregate settings. This retrospective cohort study included 116,742 contacts identified during the investigation of 2,609 TB cases diagnosed from January to December 2015. We searched the Korean National Tuberculosis Surveillance System TB registry to identify contacts that developed active TB during follow-up until May 2018. During the mean observation period of 2.9 years, 499 of 116,742 contacts (0.4%) developed new active TB. From these contacts, 404 (81.0%) developed TB within 2 years after exposure. The 2-year Kaplan-Meier cumulative risk for TB was the highest in contacts aged ≥65 years [1%; 95% confidence interval (CI), 0.8–1.3]. Contacts with LTBI who completed chemoprophylaxis exhibited a lower risk of active TB development than those without chemoprophylaxis (adjusted hazard ratio, 0.16; 95% CI, 0.08–0.29). Aggressive contact investigation is effective for the early detection and prevention of TB in congregate settings. The risk of progression to active TB among contacts with LTBI can be minimised by the completion of chemoprophylaxis.

## Introduction

Tuberculosis (TB) imposes a high global disease burden, with more than 10 million new patients and 1.6 million annual deaths worldwide^1^. The global burden of latent TB infection (LTBI) was 23.0%, amounting to approximately 1.7 billion people. WHO South-East Asia, Western-Pacific, and Africa regions had the highest prevalence and accounted for approximately 80% of those with LTBI^1,2^.

Although the incidence of TB in the Republic of Korea (ROK) has decreased from 89 per 100,000 in 2013 to 70 per 100,000 in 2017, it remains greater than that in any other country within the Organization for Economic Cooperation and Development nations^1,3^.

Generally, 5–10% individuals with LTBI develop active TB in their lifetime, with 50% developing active disease within 2 years after infection^4–6^. Thus, the management of recent contacts of patients with infectious TB is important for the overall management of TB. The World Health Organization (WHO) recommends that tests and treatments for LTBI should be prioritised for contacts of TB patients^7^.

The Korea Centers for Disease Control and Prevention (KCDC) established a TB epidemic investigation team in 2013. Between 2013 and 2017, there were 12,447 investigations with about 700,000 contacts^8^. However, follow-up research addressing TB incidence among contacts residing in areas with intermediate-level TB incidence is lacking, and most studies have addressed household contacts, with a lack of discussion regarding the TB incidence and relevant risk factors among contacts in congregate settings^9–11^.

The present study aimed to analyse the TB incidence in individuals who had contact with TB cases in congregate settings and evaluate the risk factors that influence the development of TB among these contacts.

## Results

### Cumulative TB Risk among Contacts

**Figure 1:**
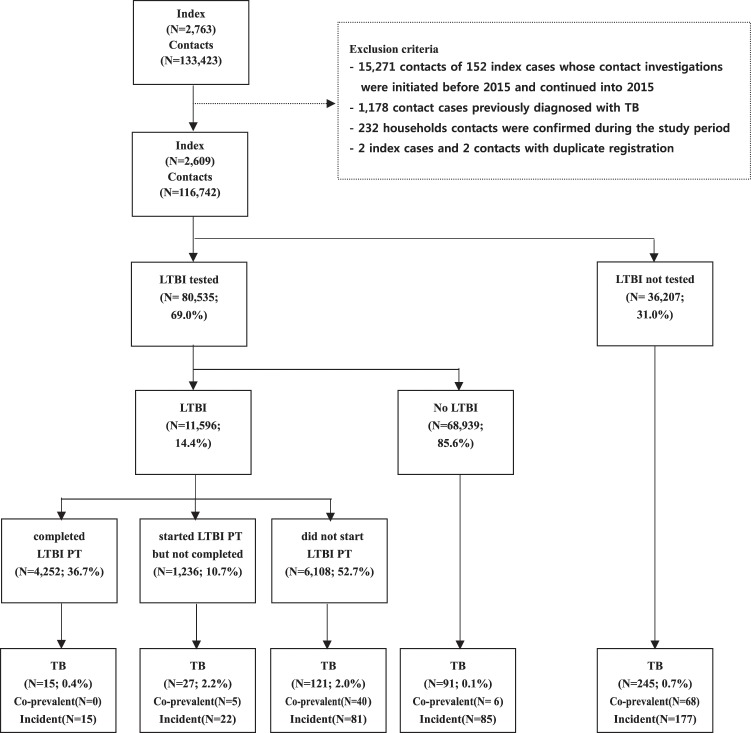
Flow chart for contacts of index cases of tuberculosis (TB) in congregate settings (January 2013 to May 2018). TB, tuberculosis; PT, preventive therapy; LTBI, latent tuberculosis infection.

**Figure 2:**
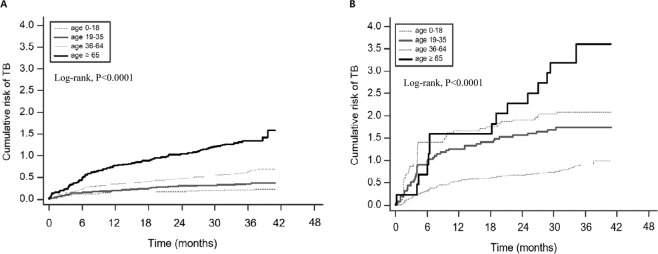
Cumulative risk of tuberculosis among contacts after notification of index cases, by age, in congregate settings. (**A**) 116,742 contacts (**B**) 11,596 contacts with latent tuberculosis infection (LTBI).

In total, 116,742 contacts of 2,609 TB cases reported between January and December 2015 were included in this study (Fig. 1). On average, the contacts were followed-up for 2.9 years until 31 May, 2018, and 499 (0.4%) developed active TB (Table 1). From these, 119 (23.8%) and 404 (81.0%) developed TB within 3 months and 2 years, respectively. The incidence of TB in contacts was 146 per 100,000 person-years; the incidence per 100,000 person-years was 414 within 3 months. The 2-year risk of TB in contacts was 0.2% [95% confidence interval (CI), 0.1–0.2] in those aged 0–18 years, 0.3% (95% CI, 0.2–0.4) in those aged 19–35 years, 0.5% (95% CI, 0.4–0.6) in those aged 36–64 years, and 1.0% (95% CI, 0.8–1.3) in those aged ≥65 years. The risk was the highest in individuals aged ≥65 years (Fig. 2A).

**Table 1:** Rates of active tuberculosis and time to diagnosis for contacts of index cases in congregate settings.

Months After Notification Index Case	Contacts with TB, No.	(Cumulative %^a^)	No. of Contacts	Person-Years of Observation, No.	Cumulative Incidence/100,000	(95% CI)	Incidence Rate/100,000^b^	(95% CI)
Total	499		116,742	340,961	427	(391.2–467.0)	146	(133.5–159.2)
0–3	119	(23.8)	116,623	28,769	102	(84.9–122.5)	414	(339.3–488.0)
4–6	85	(40.9)	116,538	28,744	73	(58.6–90.6)	296	(232.9–358.6)
7–9	50	(50.9)	116,488	28,726	43	(32.1–57.0)	174	(125.8–222.3)
10–12	48	(60.5)	116,440	30,312	41	(30.7–55.1)	158	(113.6–203.1)
13–24	102	(81.0)	116,338	116,390	88	(71.8–106.9)	88	(70.6–104.6)
25–42	95	(100)	9,958	108,020	954	(776.6–1,170.0)	88	(70.2–105.6)

TB, tuberculosis; CI, confidence interval.

^a^Calculated as the proportion of all contacts with tuberculosis (denominator, 499).

^b^Incidence rate in cases per 100,000 person-years observation.

### Cumulative TB risk among contacts with LTBI

Of the 11,596 contacts with positive test results for LTBI, 163 (1.4%) developed active TB (Table 2), with 45 (27.6%) and 138 (84.7%) diagnosed within 3 month and 2 years, respectively. The incidence of TB in these contacts was 483 per 100,000 person-years; the incidence per 100,000 person-years was 1,577 within 3 months. The 2-year risk of TB was 2.3% (95% CI, 1.2–4.3) in contacts aged ≥65 years, 1.9% (95% CI, 1.4–2.6) in those aged 0–18 years, 1.6% (95% CI, 1.1–2.2) in those aged 19–35 years, and 0.7% (95% CI, 0.5–0.9) in those aged 36–64 years. Thus, contacts aged 36–64 years exhibited the lowest risk (Fig. 2B).

**Table 2:** Rates of active tuberculosis and time to diagnosis for contacts with LTBI in congregate settings.

Months After Notification Index Case	Contacts with TB, No.	(Cumulative %^a^)	No. of Contacts	Person-Years of Observation, No.	Cumulative Incidence/100,000	(95% CI)	Incidence Rate/100,000^b^	(95% CI)
Total	163		11,596	33,731	1,406	(1,203.0–1,641.0)	483	(409.1–557.4)
0–3	45	(27.6)	11,551	2,854	390	(287.6–525.8)	1,577	(1,116.0–2,037.0)
4–6	35	(49.1)	11,516	2,843	304	(215.0–427.4)	1,231	(852.3–1,639.0)
7–9	19	(60.7)	11,497	2,837	165	(102.4–263.3)	670	(368.6–970.8)
10–12	14	(69.3)	11,483	2,990	122	(69.4–210.0)	468	(223.0–713.5)
13–24	25	(84.7)	11,458	11,470	218	(144.3–327.1)	218	(132.5–303.4)
25–42	25	(100)	3,063	10,737	816	(540.3–1,221.0)	233	(141.6–324.1)

LTBI, latent tuberculosis infection; TB, tuberculosis; CI, confidence interval.

^a^Calculated as the proportion of all contacts with tuberculosis (denominator, 163).

^b^Incidence rate in cases per 100,000 person-years observation.

### Co-prevalent TB risk among contacts

Of the 116,742 evaluated contacts, 119 (0.1%) exhibited co-prevalent TB (Table 3). The risks of co-prevalent TB were higher when index cases were male [adjusted odds ratio (aOR), 1.56; 95% CI, 1.05–2.33] and sputum smears were positive (aOR, 2.29; 95% CI, 1.45–3.59). The risks were also higher when contact occurred in healthcare facilities than when contact occurred in schools (aOR, 2.56; 95% CI, 1.62–4.06).

**Table 3:** Risk factors for co-prevalent active tuberculosis among 116,742 contacts of tuberculosis cases in congregate settings.

	Contacts Without		Contacts With		Unadjusted		Adjusted	
	Co-prevalent TB, No. (%)		Co-prevalent TB^a^, No. (%)		Odds Ratio (95% CI)	*p–value*	Odds Ratio (95% CI)	*p–value*
Total	116,623		119					
**Index factors**								
**Sex**								
Male	70,744	(99.9)	83	(0.1)	1.49 (1.01–2.21)	0.044	1.56 (1.05–2.33)	0.026
Female	45,879	(99.9)	36	(0.1)	Reference		Reference	
**Age, years**								
0–18	41,007	(99.9)	26	(0.1)	Reference			
19–35	36,147	(99.9)	36	(0.1)	1.57 (0.94–2.60)	0.079		
36–64	23,815	(99.8)	37	(0.2)	2.45 (1.48–4.04)	0.000		
≥65	15,654	(99.9)	20	(0.1)	2.01 (1.12–3.61)	0.019		
**Sputum smear status**								
Smear-positive	62,265	(99.9)	89	(0.1)	2.75 (1.80–4.21)	0.000	2.29 (1.45–3.59)	0.000
Smear-negative	53,999	(99.9)	28	(0.1)	Reference		Reference	
Unknown	359	(99.4)	2	(0.6)	10.74 (2.55–45.27)	0.001	6.80 (1.59–29.12)	0.010
**Cavities on chest radiograph**								
Cavities	29,264	(99.9)	43	(0.1)	1.67 (1.14–2.44)	0.008		
No Cavities	80,966	(99.9)	71	(0.1)	Reference			
Unknown	6,393	(99.9)	5	(0.1)	0.89 (0.36–2.21)	0.805		
**Contact factors**								
**Sex**								
Male	62,402	(99.9)	70	(0.1)	1.24 (0.86–1.78)	0.246		
Female	54,221	(99.9)	49	(0.1)	Reference			
**Age, years**								
0–18	45,103	(99.9)	30	(0.1)	Reference			
19–35	34,087	(99.9)	30	(0.1)	1.32 (0.79–2.19)	0.278		
36–64	29,183	(99.9)	40	(0.1)	2.06 (1.28–3.30)	0.003		
≥65	8,250	(99.8)	19	(0.2)	3.46 (1.94–6.15)	0.000		
**Congregate settings**								
Schools	65,740	(99.9)	41	(0.1)	Reference		Reference	
Workplaces	14,313	(99.8)	23	(0.2)	2.57 (1.54–4.29)	0.000	1.66 (0.96–2.85)	0.067
Healthcare facilities	18,364	(99.8)	36	(0.2)	3.14 (2.00–4.92)	0.000	2.56 (1.62–4.06)	0.000
Social welfare facilities	9,779	(99.9)	12	(0.1)	1.96 (1.03–3.74)	0.039	1.69 (0.88–3.26)	0.113
Others	8,427	(99.9)	7	(0.1)	1.33 (0.59–2.97)	0.483	1.09 (0.48–2.44)	0.829
**Type of contact**								
Close contact	51,225	(99.9)	61	(0.1)	1.40 (0.97–2.02)	0.073		
Casual contact	62,375	(99.9)	53	(0.1)	Reference			
Unknown	3,023	(99.8)	5	(0.2)	1.94 (0.77–4.87)	0.155		

TB, tuberculosis; CI, confidence interval.

^a^Contact diagnosed within 90 days after diagnosis of the index patient.

### Incident TB risk among contacts

Of the 116,623 evaluated contacts, 380 (0.3%) developed incident TB (Table 4). The risks of incident TB were higher when index cases were male [adjusted hazard ratio (aHR), 1.48; 95% CI, 1.15–1.90] and when cavitary lesions were present on chest radiographs (aHR, 1.97; 95% CI, 1.59–2.45]. The risks were also higher in male contacts (aHR, 1.54; 95% CI, 1.22–1.95) and contacts aged ≥65 years (aHR, 1.96; 95% CI, 1.14–3.35) than in those aged 0–18 years. Risks were higher in healthcare facilities (aHR, 3.34; 95% CI, 2.18–5.13) and social welfare facilities (aHR, 3.14; 95% CI, 1.90–5.18) than in schools. Furthermore, the risks of incident TB were higher in close contacts than in casual contacts (aHR, 1.76; 95% CI, 1.41–2.20), while they were lower in contacts with negative LTBI screening results (aHR, 0.12; 95% CI, 0.08–0.17), those who were not tested for LTBI (aHR, 0.40; 95% CI, 0.30–0.53), and those who initiated chemoprophylaxis (aHR, 0.50; 95% CI, 0.33–0.74) than in those with positive LTBI screening results who did not receive chemoprophylaxis.

**Table 4:** Risk factors for incident active tuberculosis among 116,623 contacts of tuberculosis cases in congregate settings.

	Contacts Without		Contacts With		Unadjusted		Adjusted	
	Incident TB, No. (%)		Incident TBa, No. (%)		Hazard Ratio (95% CI)	*p–value*	Hazard Ratio (95% CI)	*p–value*
Total	116,243		380					
**Index factors**								
**Sex**								
Male	70,468	(99.6)	276	(0.4)	1.70 (1.36–2.13)	0.000	1.48 (1.15–1.90)	0.002
Female	45,775	(99.8)	104	(0.2)	Reference		Reference	
**Age, years**								
0–18	40,942	(99.8)	65	(0.2)	Reference		Reference	
19–35	36,055	(99.7)	92	(0.3)	1.62 (1.18–2.23)	0.003	0.74 (0.46–1.18)	0.209
36–64	23,676	(99.4)	139	(0.6)	3.81 (2.83–5.11)	0.000	0.83 (0.51–1.36)	0.480
≥65	15,570	(99.5)	84	(0.5)	3.46 (2.50–4.79)	0.000	0.51 (0.29–0.90)	0.020
**Sputum smear status**								
Smear-positive	62,007	(99.6)	258	(0.4)	1.90 (1.53–2.36)	0.000		
Smear-negative	53,879	(99.8)	120	(0.2)	Reference			
Unknown	357	(99.4)	2	(0.6)	2.61 (0.64–10.56)	0.178		
**Cavities on chest radiograph**								
Cavities	29,106	(99.5)	158	(0.5)	2.11 (1.72–2.60)	0.000	1.97 (1.59–2.45)	0.000
No Cavities	80,759	(99.7)	207	(0.3)	Reference		Reference	
Unknown	6,378	(99.8)	15	(0.2)	0.87 (0.51–1.48)	0.624	0.93 (0.54–1.62)	0.816
**Contact factors**								
**Sex**								
Male	62,159	(99.6)	243	(0.4)	1.53 (1.24–1.89)	0.000	1.54 (1.22–1.95)	0.000
Female	54,084	(99.7)	137	(0.3)	Reference		Reference	
**Age, years**								
0–18	45,038	(99.9)	65	(0.1)	Reference		Reference	
19–35	33,999	(99.7)	88	(0.3)	1.81 (1.31–2.49)	0.000	1.14 (0.73–1.79)	0.552
36–64	29,045	(99.5)	138	(0.5)	3.36 (2.48–4.48)	0.000	0.85 (0.53–1.37)	0.513
≥65	8,161	(98.9)	89	(1.1)	7.72 (5.61–10.63)	0.000	1.96 (1.14–3.35)	0.014
**Congregate settings**								
Schools	65,631	(99.8)	109	(0.2)	Reference		Reference	
Workplaces	14,265	(99.7)	48	(0.3)	2.06 (1.46–2.89)	0.000	1.03 (0.65–1.63)	0.869
Healthcare facilities	18,217	(99.2)	147	(0.8)	4.90 (3.84–6.31)	0.000	3.34 (2.18–5.13)	0.000
Social welfare facilities	9,714	(99.3)	65	(0.7)	4.03 (2.96–5.48)	0.000	3.14 (1.90–5.18)	0.000
Others	8,416	(99.9)	11	(0.1)	0.79 (0.42–1.46)	0.456	0.60 (0.30–1.20)	0.149
**Type of contact**								
Close contact	51,019	(99.6)	206	(0.4)	1.57 (1.28–1.93)	0.000	1.76 (1.41–2.20)	0.000
Casual contact	62,215	(99.7)	160	(0.3)	Reference		Reference	
Unknown	3,009	(99.5)	14	(0.5)	1.82 (1.05–3.14)	0.031	1.64 (0.94–2.86)	0.078
**LTBI status**								
LTBI and therapy started	5,446	(99.3)	37	(0.7)	0.24 (0.14–0.42)	0.000	0.50 (0.33–0.74)	0.001
LTBI and no therapy	5,987	(98.7)	81	(1.3)	Reference		Reference	
NO LTBI	68,779	(99.9)	81	(0.1)	0.08 (0.06–0.11)	0.000	0.12 (0.08–0.17)	0.000
Not tested^b^	36,037	(99.5)	181	(0.5)	0.35 (0.28–0.45)	0.000	0.40 (0.30–0.53)	0.000

LTBI, latent tuberculosis infection; TB, tuberculosis; CI, confidence interval.

^a^Contact diagnosed within 90 days after diagnosis of the index patient.

^b^Included cases with unknown LTBI findings (n = 79).

### Incident TB risk among contacts with LTBI

Of 11,596 contacts who tested positive for LTBI, 45 were diagnosed with co-prevalent TB and 118 developed incident TB (Table 5). The risks of incident TB were higher when index cases were male (aHR, 1.91; 95% CI, 1.19–3.07) and when cavitary lesions were present on chest radiographs (aHR, 2.58; 95% CI, 1.76–3.77), while they were lower in contacts aged 36–64 years (aHR, 0.29; 95% CI, 0.15–0.57) than in those aged 0–18 years. The risks of incident TB were also lower in those who completed treatment for LTBI than in those who did not receive treatment (aHR, 0.16; 95% CI, 0.08–0.29).

**Table 5:** Risk factors for incident tuberculosis among 11,551 contacts with LTBI in congregate settings.

	Contacts Without		Contacts With		Unadjusted		Adjusted	
	Incident TB, No. (%)		Incident TBa, No. (%)		Hazard Ratio (95% CI)	*p–value*	Hazard Ratio (95% CI)	*p–value*
Total	11,433		118					
**Index factors**								
**Sex**								
Male	7,687	(98.8)	95	(1.2)	1.99 (1.26–3.15)	0.003	1.91 (1.19–3.07)	0.007
Female	3,746	(99.4)	23	(0.6)	Reference		Reference	
**Age, years**								
0–18	2,425	(98.8)	30	(1.2)	Reference			
19–35	3,491	(99.1)	32	(0.9)	0.75 (0.45–1.23)	0.261		
36–64	3,916	(98.9)	45	(1.1)	0.95 (0.59–1.51)	0.832		
≥65	1,601	(99.3)	11	(0.7)	0.56 (0.28–1.13)	0.108		
**Sputum smear status**								
Smear-positive	7,508	(98.8)	89	(1.2)	1.61 (1.05–2.44)	0.026		
Smear-negative	3,902	(99.3)	29	(0.7)	Reference			
Unknown	23	(100.0)	0	(0.0)	…			
**Cavities on chest radiograph**								
Cavities	3,648	(98.2)	67	(1.8)	2.64 (1.82–3.81)	0.000	2.58 (1.76–3.77)	0.000
No Cavities	7,128	(99.3)	49	(0.7)	Reference		Reference	
Unknown	657	(99.7)	2	(0.3)	0.43 (0.10–1.79)	0.250	0.65 (0.15–2.76)	0.566
**Contact factors**								
**Sex**								
Male	6,887	(98.9)	78	(1.1)	1.28 (0.87–1.87)	0.200		
Female	4,546	(99.1)	40	(0.9)	Reference			
**Age, years**								
0–18	2,336	(98.8)	28	(1.2)	Reference		Reference	
19–35	2,498	(98.8)	31	(1.2)	1.04 (0.62–1.74)	0.859	0.76 (0.41–1.40)	0.382
36–64	6,176	(99.3)	45	(0.7)	0.61 (0.38–0.99)	0.046	0.29 (0.15–0.57)	0.000
≥65	423	(96.8)	14	(3.2)	2.77 (1.46–5.27)	0.002	1.22 (0.52–2.88)	0.643
**Congregate settings**								
Schools	3,912	(98.8)	48	(1.2)	Reference		Reference	
Workplaces	2,849	(99.1)	25	(0.9)	0.72 (0.44–1.17)	0.195	0.57 (0.32–1.03)	0.067
Healthcare facilities	2,430	(98.6)	35	(1.4)	1.18 (0.76–1.83)	0.441	1.19 (0.66–2.16)	0.551
Social welfare facilities	1,291	(99.4)	8	(0.6)	0.50 (0.24–1.07)	0.076	0.78 (0.33–1.83)	0.571
Others	951	(99.8)	2	(0.2)	0.17 (0.04–0.71)	0.015	0.17 (0.04–0.75)	0.020
**Type of contact**								
Close contact	6,563	(98.8)	78	(1.2)	1.48 (0.99–2.20)	0.053		
Casual contact	4,337	(99.2)	35	(0.8)	Reference			
Unknown	533	(99.1)	5	(0.9)	1.17 (0.46–3.00)	0.735		
**Preventive therapy**								
Did not start	5,987	(98.7)	81	(1.3)	Reference		Reference	
Started, did not complete	1,209	(98.2)	22	(1.8)	1.33 (0.83–2.13)	0.231	0.97 (0.60–1.58)	0.920
Completed	4,237	(99.6)	15	(0.4)	0.23 (0.15–0.45)	0.000	0.16 (0.08–0.29)	0.000

LTBI, latent tuberculosis infection; TB, tuberculosis; CI, confidence interval.

^a^Contact diagnosed within 90 days after diagnosis of the index patient.

The incidence of TB in individuals treated for LTBI, those who initiated but did not complete the treatment, and those who did not receive treatment was 119 (95% CI, 58.9–179.6), 612 (95% CI, 356.4–867.9), and 460 (95% CI, 360.1–560.6) per 100,000 person-years, respectively. In contacts aged ≤35 years, the TB incidence in individuals treated for LTBI was 98 (95% CI, 30.0–165.4) per 100,000 person-years, 766 (95% CI, 364.9–1,168.0) per 100,000 person-years in those who did not complete the treatment, and 842 (95% CI, 570.6–1,113.0) per 100,000 person-years in those who were not treated. Among contacts aged ≥36 years, the TB incidence per 100,000 person-years was 159 (95% CI, 41.3–277.6), 453 (95% CI, 139.1–766.5), and 333 (95% CI, 234.9–431.9) in those who were treated, those who did not complete the treatment, and those who were not treated, respectively, with no statistically significant differences (Table 6).

**Table 6:** Incidence rate of tuberculosis among contacts treated by preventive treatment for LTBI in congregate settings.

	PT completed			PT Started, did not complete			Did not start PT		
	TB cases^a^	Person-Years of Observation, No.	Incidence Rate/100,000^b^ (95% CI)	TB cases^a^	Person-Years of Observation, No.	Incidence Rate/ 100,000^b^ (95% CI)	TB cases^a^	Person-Years of Observation, No.	Incidence Rate/ 100,000^b^ (95% CI)
Total	15	12,577	119 (58.9–179.6)	22	3,594	612 (356.4–867.9)	81	17,594	460 (360.1–560.6)
**Age, years**									
0–35	8	8,187	98 (30.0–165.4)	14	1,827	766 (364.9–1,168.0)	37	4,395	842 (570.6–1,113.0)
≥36	7	4,390	159 (41.3–277.6)	8	1,767	453 (139.1–766.5)	44	13,199	333 (234.9–431.9)

PT, preventive therapy; LTBI, latent tuberculosis infection; TB, tuberculosis; CI, confidence interval.

^a^Incident TB = TB diagnosed within 90 days after diagnosis of the index patient.

^b^Incidence rate in cases per 100,000 person-years observation.

## Discussion

In this study, we analysed the TB incidence in individuals who had contact with active TB cases in congregate settings in the ROK and sought to confirm the risk factors that influenced the development of TB in these cases. When 116,742 contacts of TB cases reported in 2015 were followed up for an average of 2.9 years, until 31 May, 2018, 499 (0.4%) had newly developed active TB. Of 499 TB cases, 421 (84.4%) were pulmonary tuberculosis and 78 (15.6%) were extra-pulmonary tuberculosis (see supplementary information). The incidence of TB in contacts was 427 per 100,000 person-years, with an incidence of 1,406 per 100,000 person-years in those who tested positive for LTBI. The overall incidence in contacts was almost seven times higher than that in the general population of the ROK in 2015 (63 per 100,000 persons)^3^, and the incidence in contacts who tested positive for LTBI was 22 times higher than that in the general population.

In contrast to our findings, studies conducted in the US and the Netherlands, which have a low TB incidence and burden, reported a higher TB incidence of 1.2%–3.5% in contacts^12–14^. Studies conducted in Taiwan^9^ and Hong Kong^10^, which have an intermediate TB burden, similar to that in the ROK, reported incidences of 0.7% and 1.7%, respectively. The relatively lower incidence found in the present study could be attributed to the fact that these previous studies^9,10,12–14^ included only close contacts of TB cases or household contacts.

Moreover, the definition of contacts in the ROK is more comprehensive than that used in other countries. Whereas the mean number of contacts per index case was 45 in this study, it was six in studies from the US^12,14^, 15 in a study from the Netherlands^13^, and three in a study from Hong Kong^10^. According to the 2015 ROK national guidelines, the entire school population was included as contacts when two or more TB cases were confirmed within 6 months or when three or more TB cases were confirmed among students in the same year. Although such aggressive contact investigations may be less efficient than investigations of targeted groups of contacts, the prevention of new active TB by finding and treating LTBI cases could be an important TB management strategy in countries with a relatively high TB incidence, such as the ROK^15^. Indeed, the TB incidence has been decreasing each year by 5% since 2011, with the number of TB cases among individuals aged <20 years exhibiting a marked decrease. The number of new TB cases among individuals aged <20 years was 1,501 in 2013, accounting for 4.2% of all cases. However, this number had decreased to 508 in 2018, accounting for 1.9% of all cases. Thus, the percentage of younger patients, who are often exposed to congregate settings, has been decreasing in particular^3^, possibly because of intervention involving contacts in congregate settings.

Of the 499 contacts who developed active TB in this study, 81.0% were diagnosed within 2 years. Of the 163 contacts with LTBI who eventually developed active TB, 84.7% were diagnosed within 2 years. A Canadian study that followed up contacts in congregate settings and household contacts for an average of 6 years reported that 86% contacts who developed active TB were diagnosed within 2 years, similar to our findings^16^. Although differing in terms of the follow-up duration and participants, recent studies, including the present one^13,14,16–18^, have reported that the 2-year incidence of TB in contacts was 63%–94.9%, which is higher than that reported in earlier studies^5,19^. According to the ROK guidelines on follow-up of contacts, follow-up chest radiography was conducted at 3 months and 9 months after exposure in contacts who were negative for LTBI. When contacts who were positive for LTBI were not treated, they underwent follow-up chest radiography every 3 months for 2 years^20^. Considering that 60.5% and 81.0% contacts developed TB within 1 and 2 years, respectively, it would be advisable to develop stratified strategies for prolonging the follow-up duration for each contact category beyond that required by the current ROK guidelines.

The Korean War has often been considered the cause of the high TB incidence in the ROK, despite the developed economic status in this country^21^. It is estimated that many Koreans were infected with TB in the poor post-war environments in the 1950s and 60 s. The LTBI rate in the ROK was 64.2% in 1960, 59.3% in 1975, 44.4% in 1990, and 33.2% in 2016^22^. The number of new TB cases aged ≥65 years has increased annually, accounting for 45.5% of all TB patients in 2018^3^. In the present study, the LTBI rate was 5.9% in individuals aged 0–18 years, 11.2% in those aged 19–35, 37.4% in those aged 36–64, and 44.5% in those aged ≥65 years (see supplementary information). The risk of incident TB was higher in those aged ≥65 years than in those aged 0–18 years, possibly because the cases were previously infected with TB and developed active TB as they aged and their immune functions decreased. In contrast, among individuals with LBTI, the risks of incident TB were lower in those aged 36–64 years than in those aged 0–18 years, opposite to the trend observed for the LTBI rate. Here, individuals aged 0–18 years were likely to have been infected with *Mycobacterium tuberculosis* relatively more recently and were more likely to develop active TB within 2 years.

The risks of co-prevalent and incident TB were higher in healthcare facilities and social welfare facilities than in schools. This may be because most contacts belonging to medical and social welfare facilities have acute or chronic conditions relative to those in other settings. In the present study, the mean age of contacts in these settings was 52.1 ± 18.3 years (see supplementary information), an age group with a relatively high prevalence of diabetes and chronic renal failure^23–25^, which are known TB risk factors. However, because we could not confirm the presence of underlying diseases in contacts, follow-up research is required.

Positive sputum smear results, identification of cavitary lung lesions on radiographs, and the presence of symptoms consistent with TB infection are known risk factors for TB in contacts^26,27^. In the present study, contacts of TB cases with cavitary lung lesions on chest radiographs exhibited greater risks of incident TB. Although the contact investigation undertaken for this study indicated that the presence of cavitary lung lesions on chest radiographs predicted the length of infectivity of index cases, radiographic examination is not currently included among the criteria for conducting investigations. Therefore, we suggest that it would be advisable to include radiography in future criteria.

In is generally accepted that 10% individuals who acquire LTBI will develop active TB in the absence of preventive therapy, with 50% developing the disease within 2 years after exposure^4,16^.

A Dutch study^28^ reported that the incidence of TB per 100,000 person-years was 187 in individuals treated for LTBI, 436 in those who did not complete treatment, and 355 in those who were not treated, similar to our findings. In the present study, among individuals aged ≥36 years, the TB incidence per 100,000 person-years was 159 in those who completed chemoprophylaxis and 333 in those who were not treated. Although the incidence was lower in those who completed chemoprophylaxis, the difference was not significant. Because the 2015 ROK national guidelines on TB management only recommend treatment for LTBI in contacts aged <36 years, testing and treatment for LTBI were not as active in contacts aged ≥36 years, which may have influenced the results. More detailed analyses on the effects of treatment for LTBI are required.

The present study had the following limitations. First, we lacked information on underlying diseases, HIV infection, diabetes, nutritional status, body mass index, and smoking, which are recognised TB risk factors, for the contacts. Second, the death of participants during the follow-up period could not be confirmed. Third, because molecular epidemiological tests for index cases and contacts were conducted only in some epidemic cases, the results were not included in the analysis. This limited analysis of the exact route of transmission. Finally, the mean follow-up duration was 2.92 years, which may be too short for the evaluation of active TB development.

In conclusion, the present study utilised complete enumeration data obtained from contact investigations conducted in the ROK in 2015 to confirm the development of TB in recent contacts within 2 years and evaluated the effects of the current contact investigations conducted in the ROK. The findings suggest that the completion of chemoprophylaxis for LTBI can lower the risk of TB development in contacts. In future, efforts to increase the contact investigation rates and improve the treatment rates for LTBI are necessary. Furthermore, long-term assessment of the effects of contact investigations is also necessary.

## Materials and Methods

### Study design and participants

This retrospective cohort study was conducted for an average 2.9 years form January 2015 to May 2018. The database for TB epidemiological investigation in congregate settings of the Korean National Tuberculosis Surveillance System (KNTSS), operated by the Korea Centers for Disease Control and Prevention, and the database for reported TB cases were used in this study. Of TB cases in congregate settings, reported to the KNTSS between January 2015 and December 2015, 2,763 cases for whom contact investigations were conducted according to the national guidelines on TB management were identified. To address potential sources of bias, contacts previously diagnosed with TB and contact investigations initiated before 2015 that continued into 2015 were excluded. The household contacts who were confirmed during the study period were also excluded. The timing of TB in contacts was defined as the time between the date on which the index TB case, present in a congregate setting, was reported and the date on which a contact of this individual was reported as an active TB case. Follow-up was continued until active TB was reported in contacts, and until May 31, 2018, in contacts who did not develop TB.

### Contact investigation

According to the Tuberculosis Prevention Act in the ROK, physicians who diagnose TB in private healthcare institutions or public health centres should report this to the public health authority through the KNTSS. TB management staff at public health centres and TB management nurses at private healthcare institutions then conduct case investigations to confirm whether the patients are associated with congregate settings and conduct contact investigation for such cases. KTNESS operates on a web - basis and consists of a patient reporting database, database for TB epidemiological investigation in congregate settings, and households contact investigation database

In accordance with the 2015 national guidelines for TB management, contact investigation was conducted when respiratory TB cases with a positive acid-fast bacilli smear test or culture test were reported to be associated with congregate settings, or when more than two TB cases were reported in the same congregate setting within 6 months, regardless of the findings in respiratory specimens^20^. On the basis of on-site investigations, contacts were classified as close or casual contacts; chest radiography and LTBI screening were prioritised for close contacts. Close contacts were those who used the same closed indoor spaces and had direct contact with the index cases for prolonged periods of times. LTBI screening involved a tuberculin skin test (TST) or an interferon-gamma release assay (IGRA). A positive TST result is defined as an induration of ≥10 mm (≥5 mm in newborns that had not yet received the BCG vaccine). The IGRA was performed using the QuantiFERON-TB Gold In-tube (QTF) test, and a value of 0.35 international units or more was deemed positive^20^.

### Definitions and risk factors

‘Index patient’ was defined as the first person with confirmed TB within the congregate settings. When contacts were reported to have developed TB within 90 days after the initial report of TB in the index case, the disease was considered to be ‘co-prevalent’. When contacts were reported to have developed TB after 90 days of the report of active TB in the index case, the disease was considered ‘incident’^29^. Risk factors related to the index cases included sex, age, sputum smear microscopy results, and chest radiograph results, while those for contacts included sex, age, the congregate setting, the type of contact, the presence of LTBI, and treatment for LTBI. Congregate settings were categorized into school, workplace, healthcare facility, social welfare facility, and others.

### Study population

Overall, 133,423 contacts of 2,763 index cases were extracted from the KNTSS.

The following participants were excluded: 15,271 contacts of 152 index cases whose contact investigations initiated before 2015 and continued into 2015, 1,178 contact cases previously diagnosed with TB, 232 household contacts confirmed during the study period, 2 index cases and 2 contacts with duplicate registration. The final sample included 116, 742 contacts of 2,609 index cases (Fig. 1). Of the index cases, 2,594 (99.4%) were pulmonary tuberculosis.

### Ethics approval and consent to participate

The need for written informed consent from participants was waived, based on the Korean Infectious Disease Control and Prevention Act (No.4). This study was conducted in accordance with Korean Infectious Disease Control and Prevention Act and Tuberculosis Prevention Act with permission of KCDC. The study design was approved by the Institutional Review Board (IRB) of KCDC. As this is a retrospective study on the existing data on subjects and based on the Korean Infectious Disease Control and Prevention Act (No.4), we received confirmation of the written consent exemption from IRB.

### Statistical analyses

The Kaplan–Meier method and log-rank tests were used to compare TB incidences according to the contacts’ age. Demographic, laboratory, and clinical determinants (both index cases and contact-related cases) of co-prevalent TB were identified using logistic regression, while those of incident TB were analysed using Cox proportional hazards regression. Multivariate analyses with backward elimination were conducted for the sex and age of index cases and contacts as well as variables with a p-value of <0.05 in univariate analyses. Statistical significance was identified with a 95% confidence interval and a P-value < 0.05. All statistical analyses were performed using SAS version 9.4 (SAS Institute Inc) and Epi-infotm (CDC, Atlanta, GA, USA).

## Supplementary information

Supplementary Information.

## Data Availability

All data extracted in this study are included in this article.
