# Supplementary material for: Risk of active tuberculosis development in contacts exposed to infectious tuberculosis in congregate settings in Korea
Source: Sci Rep. 2020 Jan 28;10:1306. doi: 10.1038/s41598-020-57697-1 (PMC6987175; doi:10.1038/s41598-020-57697-1)
Supplement: Supplementary file 1 — Supplementary Information. [file 41598_2020_57697_MOESM1_ESM.docx]

# Supplementary Information

***Risk of active tuberculosis development in contacts exposed to infectious tuberculosis in congregate settings in Korea***

Shin Young Park^1^, Sunmi Han^1^, Youngman Kim^1^, Jieun Kim^1^, Sodam Lee^1^, Jiyeon Yang^1^, Un-Na Kim^1,*^, Mi-sun Park^1,*^

(*corresponding author)

^1^ Division of TB Epidemic Investigation, Korea Centers for Disease Control and Prevention, 28159 Osong Health Technology Administration Complex, 187, Osongsaengmyeong 2-ro, Osong-eup, Heungdeok-gu, Cheongju-si, Chungcheongbuk-do, Republic of Korea.

**Supplemental Table 1.** Demographic and clinical characteristics of tuberculosis index cases

| **Characteristics** | Number (%) |
| --- | --- |
| **Total** | **2,609** (100.0) |
| Sex |  |
| Male | 1,498 (57.4) |
| Female | 1,111 (42.6) |
| Age |  |
| 0–18 | 311 (11.9) |
| 19–35 | 976 (37.4) |
| 36–64 | 806 (30.9) |
| ≥ 65 | 516 (19.8) |
| Type of tuberculosis |  |
| Pulmonary tuberculosis | 2,578 (98.8) |
| Extra-pulmonary tuberculosis | 31 (1.2) |
| Congregate settings (years, mean ± SD) |  |
| Schools (23.4±10.6) | 723 (27.7) |
| Workplaces (39.3±11.7) | 827 (31.7) |
| Healthcare facilities (59.1±22.6) | 530 (20.3) |
| Social welfare facilities (73.7± 18.5) | 316 (12.1) |
| Other (31.8±14.8) | 213 (8.2) |
| Sputum smear status |  |
| Smear-positive | 1,749 (67.0) |
| Smear-negative | 853 (32.7) |
| Unknown | 7 (0.3) |
| Cavities on chest radiograph |  |
| Cavities | 754 (28.9) |
| No Cavities | 1,692 (64.9) |
| Unknown | 163 (6.2) |

The mean age of the 2,609 index cases was 42.5 (±23.0) years.

**Supplemental Table 2**. Demographic and clinical characteristics of congregate settings contacts of the index cases

| **Characteristics** | **Overall** | **Contacts**  **without active tuberculosis** | **Contacts**  **with active**  **tuberculosis** |
| --- | --- | --- | --- |
|  | Number **(**%) | Number **(**%) | Number **(**%) |
| **Total** | **116,742** (100.0) | **116,243** (100.0) | **499** (100.0) |
| Sex |  |  |  |
| Male | 62,472 (53.5) | 62,159 (53.5) | 313 (62.7) |
| Female | 54,270 (46.5) | 54,084 (46.5) | 186 (37.3) |
| Age |  |  |  |
| 0–18 | 45,133 (38.7) | 45,038 (38.8) | 95 (19.0) |
| 19–35 | 34,117 (29.2) | 33,999 (29.2) | 118 (23.6) |
| 36–64 | 29,223 (25.0) | 29,045 (25.0) | 178 (35.7) |
| ≥ 65 | 8,269 (7.1) | 8,161 (7.0) | 108 (21.6) |
| Congregate settings (years, mean ± SD) |  |  |  |
| Schools (20.0±9.7) | 65,781 (56.3) | 65,631 (56.5) | 150 (30.1) |
| Workplaces (37.7±10.8) | 14,336 (12.3) | 14,265 (12.3) | 71 (14.2) |
| Healthcare facilities (52.1±18.3) | 18,400 (15.8) | 18,217 (15.7) | 183 (36.7) |
| Social welfare facilities (60.2±19.6) | 9,791 (8.4) | 9,714 (8.4) | 77 (15.4) |
| Other (24.0±11.9) | 8,434 (7.2) | 8,416 (7.2) | 18 (3.6) |
| Type of contact |  |  |  |
| Close contact | 51,286 (43.9) | 51,019 (43.9) | 267 (53.5) |
| Casual contact | 62,428 (53.5) | 62,215 (53.5) | 213 (42.7) |
| Unknown | 3,028 (2.6) | 3,009 (2.6) | 19 (3.8) |
| LTBI screening result |  |  |  |
| Positive | 11,596 (9.9) | 11,433 (9.8) | 164 (32.7) |
| Negative | 68,860 (59.0) | 68,773 (59.2) | 87 (17.4) |
| Unknown | 79 (0.1) | 75 (0.1) | 4 (0.8) |
| Not tested | 36,207 (31.0) | 35,962 (30.9) | 245 (49.1) |

The mean age of the 116,742 contacts was 30.9 (±19.5) years.

**Supplemental Table 3**. The rate of latent tuberculosis infection in contacts.

|  | No. of individual tested | No. of LTBI | LTBI rate |
| --- | --- | --- | --- |
| **Total** | **80,535 (100.0)** | **11,644** | **14.4** |
| Age (year) |  |  |  |
| 0–18 | 40,210 (49.9) | 2,385 | 5.9 |
| 19–35 | 22,691 (28.2) | 2,542 | 11.2 |
| 36–64 | 16,650 (20.7) | 6,231 | 37.4 |
| ≥ 65 | 984 (1.2) | 438 | 44.5 |
| Congregate settings |  |  |  |
| Schools | 52,150 (64.8) | 3,984 | 7.6 |
| Workplaces | 10,432 (13.0) | 2,886 | 27.7 |
| Healthcare facilities | 7,353 (9.1) | 2,469 | 33.6 |
| Social welfare facilities | 3,722 (4.6) | 1,301 | 35.0 |
| Other | 6,878 (8.5) | 956 | 13.9 |

**Supplemental Table 4**. Newly developed type of tuberculosis among contacts.

| **Type of tuberculosis** | Number (%) |
| --- | --- |
| **Total** | 499 (100.0) |
| Pulmonary tuberculosis | 421 (84.4) |
| Extra-pulmonary | 78 (15.6) |
| Tuberculous Pleuritis | 39 (50.0) |
| Tuberculous peripheral lymphadenopathy | 23 (29.5) |
| Tuberculosis of intestines, peritoneum and mesenteric glands | 9 (11.5) |
| Tuberculous Meningitis | 2 (2.6) |
| Tuberculosis of bones and joints | 2 (2.6) |
| Tuberculosis of other specific organs | 2 (2.6) |
| Tuberculosis of skin and subcutaneous tissues | 1 (1.3) |

| **A**  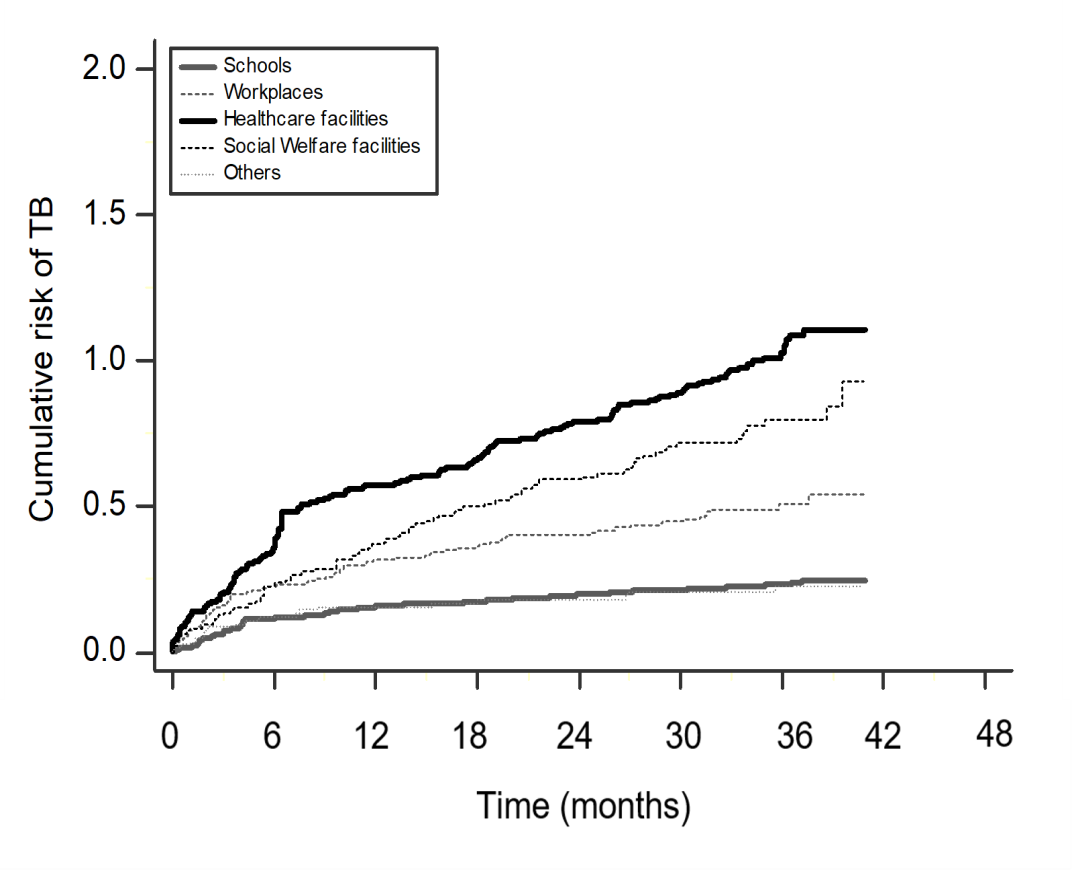  Log-rank, P<0.0001 | **B**  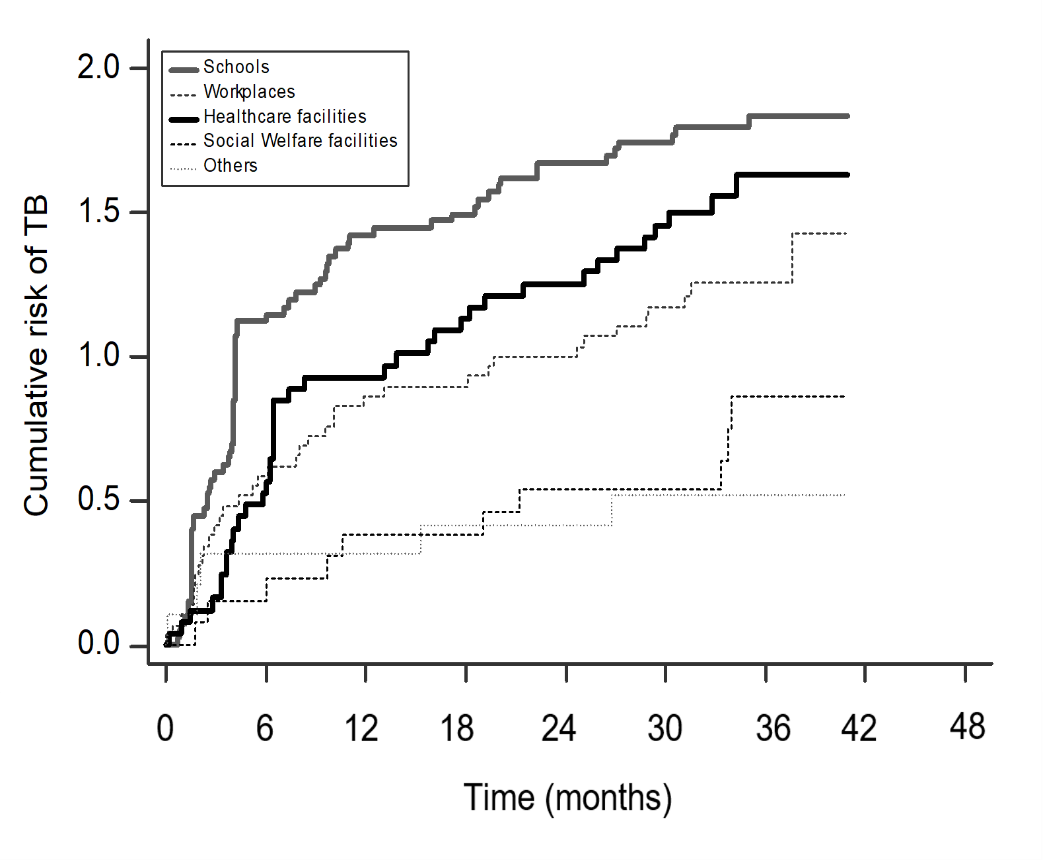  Log-rank, P<0.01 |
| --- | --- |
| **Supplemental Figure 1.** Cumulative risk of tuberculosis among contacts after notification of index cases, by congregate settings  (A) 116,742 contacts (B) 11,596 contacts with latent tuberculosis infection (LTBI) | |
